# Patients’ Data Management System Protected by Identity-Based Authentication and Key Exchange

**DOI:** 10.3390/s17040733

**Published:** 2017-03-31

**Authors:** Alexandra Rivero-García, Iván Santos-González, Candelaria Hernández-Goya, Pino Caballero-Gil, Moti Yung

**Affiliations:** 1 Department of Computer Engineering and Systems, University of La Laguna, 38206 Tenerife, Spain; ariverog@ull.edu.es (A.R.-G.); jsantosg@ull.edu.es (I.S.-G.); mchgoya@ull.edu.es (C.H.-G.); 2 Computer Science Department, Snapchat and Columbia University, New York, NY 10027, USA; moti@cs.columbia.edu

**Keywords:** identity-based cryptosystem, identity-based authentication and key exchange, mHealth, keyed-hash message authentication code, Android, NFC

## Abstract

A secure and distributed framework for the management of patients’ information in emergency and hospitalization services is proposed here in order to seek improvements in efficiency and security in this important area. In particular, confidentiality protection, mutual authentication, and automatic identification of patients are provided. The proposed system is based on two types of devices: Near Field Communication (NFC) wristbands assigned to patients, and mobile devices assigned to medical staff. Two other main elements of the system are an intermediate server to manage the involved data, and a second server with a private key generator to define the information required to protect communications. An identity-based authentication and key exchange scheme is essential to provide confidential communication and mutual authentication between the medical staff and the private key generator through an intermediate server. The identification of patients is carried out through a keyed-hash message authentication code. Thanks to the combination of the aforementioned tools, a secure alternative mobile health (mHealth) scheme for managing patients’ data is defined for emergency and hospitalization services. Different parts of the proposed system have been implemented, including mobile application, intermediate server, private key generator and communication channels. Apart from that, several simulations have been performed, and, compared with the current system, significant improvements in efficiency have been observed.

## 1. Introduction

One of the most innovative paradigms of the last years in the healthcare sector is the integration of mobile devices in the practice of medicine and public health, known as mHealth. Its significance stems from the flexibility provided by the use of mobile devices. However, the potential security problems that arise from the use of mobile devices and their wireless interface must be carefully addressed due to the strict privacy requirements of medical data. In the work [1], a few recommendations to solve some of these problems are explained in detail.

This paper presents a secure and distributed system for the management of patients’ data in emergency and hospitalization services with the primary goal of improving efficiency and security. In particular, several cryptographic protocols are used to protect the confidentiality of the communications and the access control to patient records.

The risk of patient misidentification is an issue to which health authorities pay a lot of attention, in order to try to avoid dangerous consequences such as medication errors, incorrect surgical procedures, etc. [2]. In spite of that, statistical data on this subject are worrying. For example, in the UK, the National Health Service received more than 24,000 statements on misidentifications of patients in 2006–2007 [3].

One of the bases of the proposal is the use of Near Field Communication (NFC) [4], specifically NFC wristbands, for automatic patient identification. Unlike other technologies such as Radio Frequency IDentification (RFID) [5], Bluetooth or Wi-Fi [6], NFC is not oriented to continuous data transmission because it requires a temporal contact between the devices that interact in order to allow the exchange of information in a quick and timely manner. Although at first glance the distance factor for transmitting information may seem a limitation, it is actually a key point of this technology. The need for proximity between devices limits the types of attacks that can be launched. In addition, not requiring pairing between devices facilitates its use by medical staff.

Apart from the NFC wristbands , the other main components of the system are: a mobile device associated to each member of the medical staff, the intermediate server that hosts a web service, an NFC reader and writer for allocating wristbands once patients have been identified, and a second server in charge of producing information to protect the exchange of information.

This work is organized as follows. Section 2 provides some related works while Section 3 gives a general description of the proposed system. The topic of patient identification through NFC tags and keyed-Hash Message Authentication Code (HMAC) schemes in emergency and hospitalization services is dealt with in Section 4. The protection of communications between the medical staff and the intermediate server through Identity-Based Authentication and Key Exchange is proposed in Section 5. A brief security analysis is provided in Section 6. The implementation of parts of the proposal and simulations of the system are explained in Section 7. Finally, a few conclusions and future works close the paper.

## 2. Related Works

Recently, different solutions to solve specific problems in the management of the patients’ data have been proposed. The security in healthcare applications is one of the most important points, even more when wireless sensor networks are used [7]. The specific case of wireless body area networks is very useful in healthcare, as shown in [8], where sensors are wearable devices that allow for obtaining, computing and distributing information about patients.

The data shared by these networks are usually stored as electronic health records so that the protection of the privacy of these records is very important. In the work [9], a system using an attribute-based infrastructure is proposed to preserve the privacy of the data. In that system, the registration of patients and doctors is performed with a username and a password, and the identification of the patient is a private user-index.

The so-called Personal Health Record (PHR) facilitates the management of medical records in a centralized way. One of the improvements obtained with the use of PHR is that patients can analyse their own information. In the work [10], a proposal of this kind of system is proposed based on the use of PHR in cloud computing, where an attribute-based encryption protocol is used to obtain the information. However, in that paper, the identification of patients and authentication of doctors are not explained.

Sharing medical care information in a secure way is proposed in the work [11], thanks to the use of role-based secure messaging services. The main problem of this approach is that a specific e-mail provider is used, and that the authentication is based on external tools.

In the paper [12], the authors introduce a mutual authentication scheme to improve the security of automatic systems for medication through RFID bracelets and cryptography based on elliptic curves. However, the used protocol does not provide a mechanism for session key generation to protect confidentiality.

There are proposals for medical systems that base their operation on the use of NFC bracelets. In the work [13], an example is shown where it is assumed that every day each patient uses a bracelet. If there is an emergency that requires checking of medical data, these are read using a mobile application. A disadvantage of that application is that any user who has the application could perform an information query because there is no security mechanism implemented.

In the work [14], a system for the management of medical staff rounds is described that uses NFC wristbands and mobile devices. However, it does not specify whether security services are deployed or not.

The authors of the publication [15] developed an attack on a mutual authentication system based on RFID tags defined in [16]. Specifically, they show that it is possible to trace the tags so the protection of patient privacy is not guaranteed. At the same time, a new protocol is defined that solves the privacy problem and improves the efficiency of the system. An authentication scheme for the RFID tag is introduced, but it does not take into account the security of RFID readers. Apart from this, the server used to manage the information is also in charge of generating and storing the keys.

The proposal presented here includes automatic patient identification, mutual authentication between server and medical staff, and protection of confidentiality in the communications between the mobile device and the server. Confidentiality between the mobile device and wristband is warranted by the need for proximity to establish the NFC connection. Thus, this integration of security tools provides a robust solution that improves patient management and daily routine of medical staff.

## 3. System Overview

In most current health systems, when a patient arrives at a hospital, the fist step that the staff must do is to identify her/him. Patient identification is usually performed through the verification of a health identification card. Then, the patient is evaluated by a healthcare staff member who analyses the information collected during admission and adds the results of new assessments if required. Afterwards, the patient may be seen by a specialist. Before each one of these actions, the process of patient identification must be repeated. This current system has several drawbacks. Doctors must check the patient record before assisting her/him. In order to do it, depending on the particular case, they can make such a consultation through printed documentation or by using a computer. If paper documentation is used, it is usually generated as a batch for a set of patients. For example, three medical records may be printed at a time so that a doctor can check and attend those three patients one after the other. Once they are attended, the doctor should leave the records and repeat the process with a new group of patients. This arrangement produces heterogeneous information because some data may be updated on computers while other data are kept in printed format.

In addition, updates made by doctors are not changed in the central system in real time. In this approach, health workers have to deal with a lot of documentation, which leads to consuming considerable time and resources. On the other hand, each member of the medical staff has to visit several patients at each turn, which may generate wrong patient identifications, with serious consequences in some cases.

A solution to these issues is described in this work, which consists in the implementation of a secure system based on NFC wristbands and mobile devices that allow for eliminating patient misidentification and rationalizing the use of time in patient care.

The proposal involves substantial changes with respect to the traditional system. When patient identification is performed for the first time, an NFC wristband is assigned to him/her. Specifically, the NTAG21x ICs [17] wristband, which follows the pattern set by the NFC Forum (association that regulates the NFC standards) [18] is recommended here. This wristband will not be used to store any sensitive patient data. The only data stored on the wristband is an identifier assigned by the server. This identifier is generated through a process that will be discussed below. Such a generation takes into account the physical identifier of the wristband (similar to the Media Access Control (MAC) address number of computers) together with the patient record number. Note that this information will be written on the wristband in a completely secure way.

This wristband can be deployed both in the inpatient and emergency areas. It can be even assigned before the patient arrives to hospital, in the ambulance, where the identification and the writing procedures could be done through a mobile phone.

The data stored in the wristband allow any member of the medical staff with the right permissions access to the patient record identifying the patient with the simple gesture of bringing a mobile device close to the wristband. Thanks to the use of wristbands, the system prevents confusion when identifying patients and increases efficiency in the development of medical tasks. In addition, wristbands are fully recyclable, so when a patient leaves the hospital, its wristband is reset to be used by another patient.

The system is designed to work with two separated servers, here referred to as the intermediate server and second server. On the one hand, the intermediate server manages access permissions to patients’ data on the basis of medical staff shifts. On the other hand, the second server uses a Private Key Generator (PKG) to manage the information related to keys.

The use of two different physical servers is proposed to add a new security layer in the management of the keys. With this separation, different firewalls can be added to each server independently and different secure rules can be applied in the communications between them. Specifically, the limitation of the communication of the private key server to intra-communication (intranet communications) is advisable. In other words, the communication of the PKG with the extranet can be denied and just some interactions with the intermediate server can be allowed through, for example, an intranet. In this way, if the intermediate server is corrupted by an attacker, both the private key generator and the server keys should not be involved. Although having two servers is more expensive than having just one, since nowadays the value of a dedicated server in the cloud is about $50 a year, we consider that this is a very low value when compared with the security that it brings to the proposed system.

The protection of patients’ data is a paramount objective in the healthcare environment. This is why security is one of the pillars of the described solution. A keyed-Hash Message Authentication Code (HMAC) is applied for automatic patient identification, and IDentity-based (ID-based) cryptography is used to protect confidentiality of patient records.

The security of the communication between doctors and the intermediate server is based on an ID-based Authentication and a Key Exchange (AKE) scheme that provides mutual authentication between doctors and the server through a PKG. Next, the details on how these security tools are used in the proposed framework are included.

## 4. Automatic Patient Identification

As aforementioned, when a patient arrives at a hospital, the first step is the identification through his/her credentials. After that, in the proposal, an NFC wristband is assigned to him/her so that each patient is identified through an HMAC generated by the intermediate server by using the physical identifier of the wristband and the patient record number. If a patient does not have a medical record in the system, it is automatically created with some basic fields, such as name, age, country, etc.

**Figure 1:**
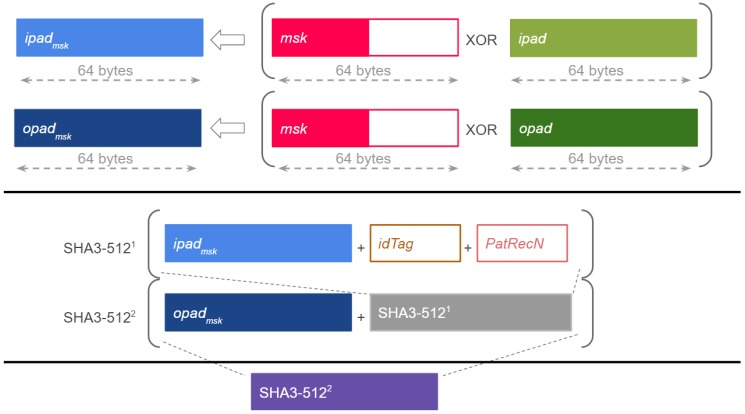
Keyed-hash message authentication code Generation.

The generation of the HMAC can be seen in Figure 1. The system sends the physical identifier of the wristband to the intermediate server, and two 64-byte arrays denoted as ipad and opad are generated as in the work [19], where some default values are assigned to them during the initialization of the HMAC generation. New arrays denoted by $ipad_{msk}$ and $opad_{msk}$ are generated through an bit level exclusive OR operation on ipad and opad respectively, and the master secret key (msk). Then, with the physical identifier of the wristband Tag (idTag) and the Patient Record Number (PatRecN), the system uses a SHA3-512 hash function [20] to generate the HMAC value. Firstly, the hash function is applied to the concatenation of $ipad_{msk}$, idTag and PatRecN. Secondly, the output of this hash function concatenated with the $opad_{msk}$ is the input to another hash function so that the HMAC is the final result. This output is stored in the NFC wristband to be used as patient identifier.

When trying to access to a patient data, his/her NFC wristband must be read through a doctor’s device, which sends the data obtained from the wristband, corresponding to the physical identifier of the wristband and the HMAC, to the server. The server verifies the authenticity of the bracelet and the doctor’s access permissions. If the verification is positive, the authentication protocol described later is used each time a member of the medical staff needs to access to patients’ data.

The protection of communications is achieved through an ID-based scheme. In this type of public key cryptography schemes, any text can act as a valid public key with a PKG. The main reason to choose this approach for the proposal is the simplification of management because in this way it is not necessary to define a public key infrastructure. Furthermore, an ID-based scheme was chosen because of its low computational complexity and its efficiency in terms of memory and usability.

**Figure 2:**
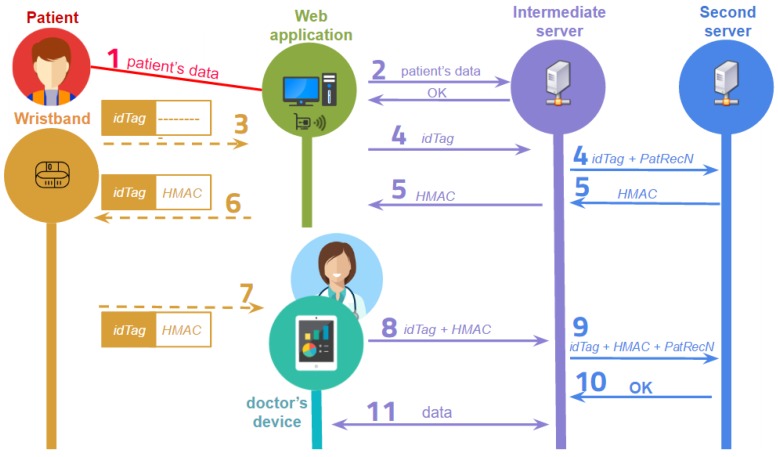
Medical record consultation.

The description of all the steps of the communication flow during a medical record consultation between the participants in the system is included below (see Figure 2):

1. A member of the hospital admission staff receives the basic patient’s data.
2. This patient’s information is sent to the intermediate server. The identification of the patient is analysed at the server. If the patient is registered in the system, the server stores any new information and the system sends the verification to the web application. Otherwise, the server generates a new user identification and stores the corresponding data.
3. The assignation of a wristband to a patient starts with the reading of the physical identification of the tag idTag through an NFC reader.
4. The idTag is sent from the web application to the intermediate server, which links the idTag with the patient’s medical record of number PatRecN and sends these values to the PKG in the second server.
5. The PKG generates the HMAC value with idTag, PatRecN and the pre-calculated values $ipad_{msk}$ and $opad_{msk}$. The HMAC value is sent to the intermediate server, which sends it to the web application.
6. The HMAC value is stored in the NFC tag of the patient’s wristband.
7. When a doctor wants to identify a patient, he/she has to touch the NFC wristband with his/her mobile device to read both idTag and HMAC.
8. The stored values are sent from the mobile device to the intermediate server where the wristband is identified. The medical record is then loaded.
9. The intermediate server sends to the second server idTag, HMAC and PatRecN.
10. The PKG analyses and verifies the association, and the result of this verification is sent to the intermediate server.
11. If the HMAC verification is right, the server sends the medical record values to the mobile device where the doctor can read, edit or add data. The values corresponding to a patient can be modified until a new patient’s wristband is read.

## 5. Secure Communication

Communications between the mobile device of each member of the medical staff and the intermediate server are encrypted with an ID-based scheme.

A crucial element of the proposal is the Private Key Generator in the second server because it is in charge of generating private keys for medical staff. The identifier used in the system for each member of the medical staff is the corresponding number of registered medical practitioner. Specifically, the system is based on the proposal described in [21], but adapted to a more secure infrastructure where the PKG and the intermediate server are separated.

**Figure 3:**
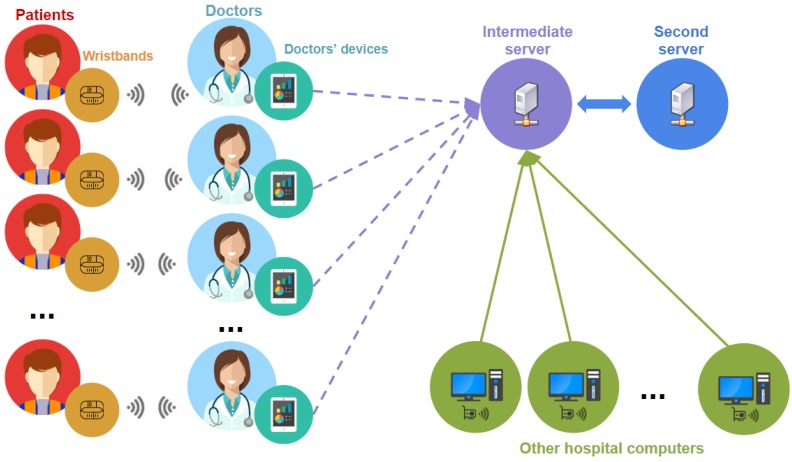
System’s communication flow.

As seen in Figure 3, on the one hand, there are different devices assigned to doctors, which are smartphones or tablets with NFC reader and Wi-Fi. On the other hand, each patient has an NFC wristband. The intermediate server is the controller of the communication between the medical staff and the PKG. In particular, the intermediate server has a public Application Programming Interface (API) for doctors’ communication and other hospital computers and a private API for communication with the PKG. Finally, the PKG is in charge of the authentication and verification of each communication. This is why server keys are stored in the PKG.

The protection of communications is achieved through an ID-based scheme. The first approach of this type of scheme was proposed by Shamir [22] in 1985 to avoid certificate management in asymmetric key systems. In this public key cryptography schemes, any text can be used as a valid public key. Specifically, the public key is usually extracted from some user’s identity information, such as name, email or health identification. Following Shamir’s idea, Boneh and Franklin proposed a practical ID-based encryption system [23] based on the Weil pairing. Starting from this proposal, multiple ID-based schemes have been introduced. Some basic ID-based schemes may be identified depending on the type of security service to be implemented: ID-based encryption schemes [24,25,26], ID-based signature schemes [27,28], ID-based signcryption schemes [29,30], ID-based group key exchange protocols [31,32] and ID-based AKE [33,34].

In the proposed system, mobile devices are used to manage patients’ information. These devices have energy and computing capability limitations, so they should not depend on heavy cryptographic computations. Taking this into account, some protocols based on a client-server paradigm have been applied. One of the most used techniques to reduce the online cost is offline pre-computation. In the offline pre-computation used in this proposal, a few random values called ephemeral secrets are required to perform some operations in advance. These values are stored in the memory of the mobile device until the system requires them in the online step.

The attack called Ephemeral-Secret-Leakage (ESL) [35,36] might be launched in the online step. In order to be resistant to ESL attacks, the present proposal uses an ID-based AKE protocol that does not use bilinear pairings. Specifically, the scheme is based on the ESL-secure ID-based AKE protocol [21] that uses an ESL-secure signature scheme [37] to manage the client-to-server authentication and the Tate pairing [38], which is faster than the basic Weil pairing [39].

The notations used within this paper are introduced below:

- *G*, $G_{T}$ : cyclic groups;
- $P,P^{\prime }$ : generators of the group *G*;
- $\hat{e}$ : a bilinear map from G×G to $G_{T}$;
- msk : randomly selected master secret key;
- mpk : master public key;
- ID : IDentification of a registered medical practitioner;
- $pgk_{ID}$ : private group key of the medical practitioners with ID;
- $H_{1},H_{2},f$ : hash functions;
- ${\left\{0,1\right\}}^{n}$ : space of all n-length binary vectors;
- ${\left\{0,1\right\}}^{*}$ : space of all binary strings of any length;
- $G^{*}$ : multiplicative group *G*\{0};
- $x\overset{r}{\leftarrow }S$ : an element *x* is randomly selected from a set *S*;
- || : concatenation;
- == : comparison.

Next, the mathematical basis used in the system is described.

Considering two cyclic groups (G,+) and $\left(G_{T},\cdot \right)$ of the same prime order *q*, there is a symmetric bilinear map pairing $\hat{e}:G\times G\to G_{T}$ with the following properties:

- Bilinear: ∀P,Q∈G and ∀a,b∈Z, $\hat{e}\left(aP,bQ\right)=\hat{e}{\left(P,Q\right)}^{ab};$
- Non-degenerate: $\exists P_{1},P_{2}\in G$ that $\hat{e}\left(P_{1},P_{2}\right)\neq 1$. This means that if *P* is generator of *G*, then $\hat{e}\left(P,P\right)$ is a generator of *Q*;
- Computable: There exists an efficient algorithm to compute $\hat{e}\left(P,Q\right),\forall P,Q\in G.$

Let a cyclic group (G,+) have prime order *q* and $P^{\prime }$ be a generator of *G*; then, the following mathematical assumption based on the so-called Elliptic Curve Diffie-Hellman (ECDH) [40] problem can be considered. Given $P^{\prime },aP^{\prime },bP^{\prime }\in G$ for unknown $a,b\in Z_{q}$, the ECDH problem consists of computing $abP^{\prime }$. No probabilistic polynomial time exists to allow an adversary to compute $abP^{\prime }$ with a non-negligible probability.

Two different types of hash functions are used.

On the one hand, two map-to-point hash functions:

$$H_{1}:{\left\{0,1\right\}}^{*}\to G^{*},H_{2}:{\left\{0,1\right\}}^{*}\to G^{*}.$$

On the other hand, a one-way hash function:

$$f:{\left\{0,1\right\}}^{*}\to {\left\{0,1\right\}}^{n},$$

where the size of the message is defined by *n*.

We assume that to concatenate a point *P* to a number N, the coordinates $\left(P_{x},P_{y}\right)$ of the point *P* are concatenated separately so that P||N is equivalent to $P_{x}\left|\right|P_{y}\left|\right|N$.

The four steps needed for the ID-based AKE scheme are: Setup, Extract, Mutual Authentication and Session Key Generation.

**Figure 4:**
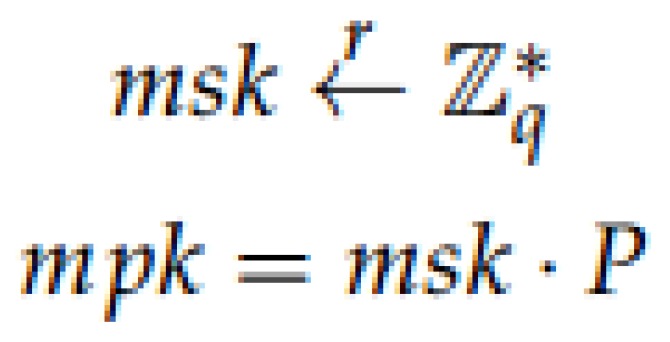
Setup phase.

**Figure 5:**
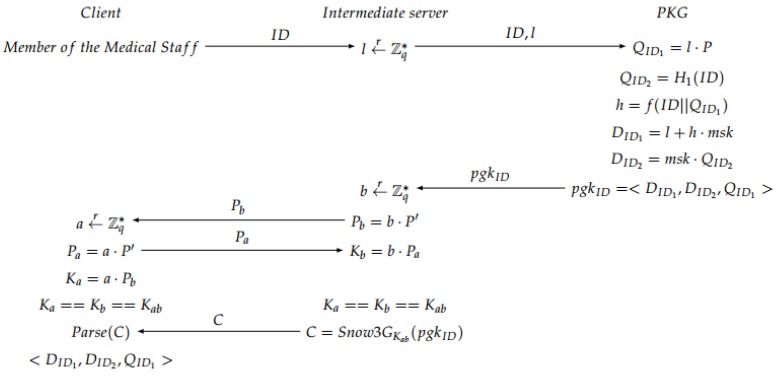
Extract phase.

**Figure 6:**
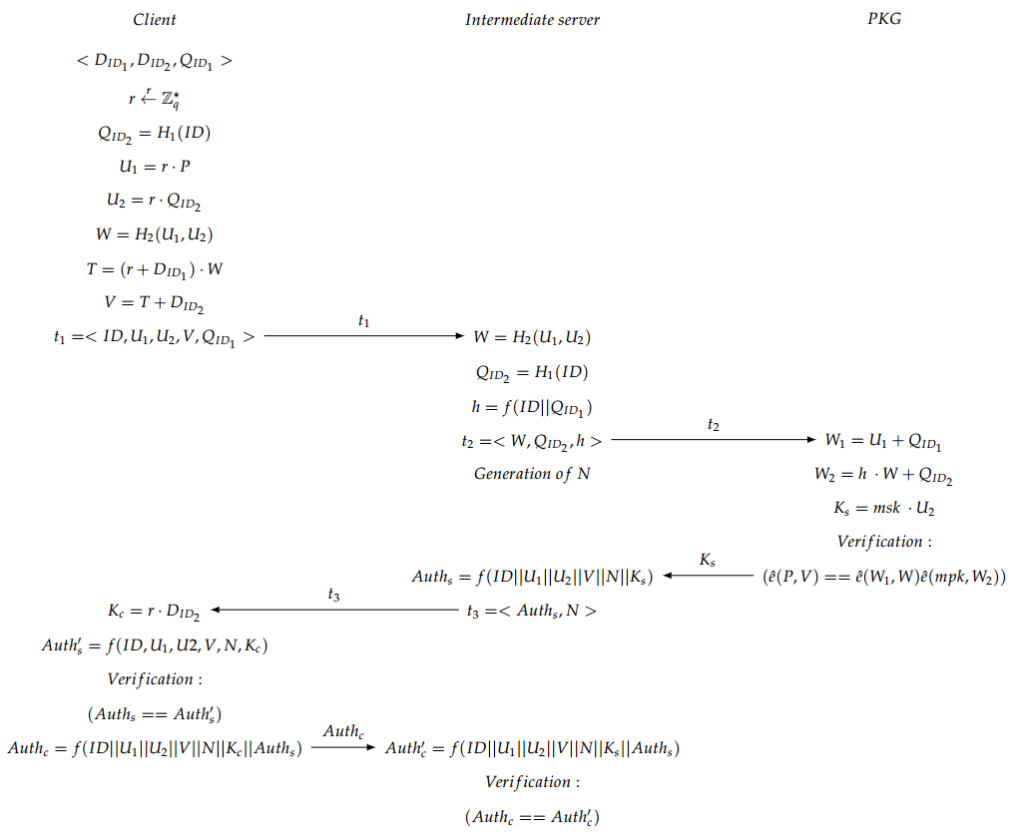
Mutual authentication phase.

**Figure 7:**
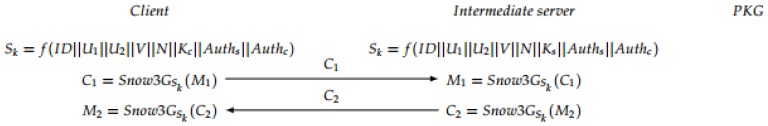
Session key generation.

- Setup: The initial parameters are established and the PKG in the second server generates the master public key mpk and the master secret key msk. For that, a prime *q* based on some private data k∈Z, two groups *G* and $G_{T}$ of order *q* and a symmetric bilinear pairing map $\hat{e}:G\times G\to G_{T}$ are selected. P∈G is randomly chosen and the hash functions $H_{1}$, $H_{2}$ and *f* are used (see Figure 4).
- Extract: The private group key for each member of the medical staff based on its ID is generated. The intermediate server generates a random number *l*, and sends it together with ID to the second server. Then, the PKG computes different values to obtain a private group key $pgk_{ID}$, which is calculated taking into account the master private key mpk. Finally, in the intermediate server, a secure channel is created based on an ECDH to share the private information with the doctor. A session key is generated to encrypt the information, obtaining an encrypted message *C* with a Snow 3G stream cipher algorithm [41] (see Figure 5).
- Mutual Authentication: Some offline computations are performed by the client to be able to perform mutual authentication. After the extract phase, the client generates and sends to the server a 3-tuple. Then, the server parses the tuple to obtain the values, and generates some new parameters. Afterwards, the server sends all the information to the PKG, who is in charge of the verification of the bilinear map pairing. If the verification is OK, the PKG sends a parameter to the server, otherwise a call “close” is sent back to the server to finish the communication. If the verification was OK, the server generates a tuple, which is sent to the client, which authenticates the server. If this authentication is OK, the client generates a parameter for its own authentication against the server and sends it back to the intermediate server, which verifies the user authentication (see Figure 6). If everything is OK, both client and server continue to the next step, which is the session key generation.
  Note that in the verification step carried out in the second server, the PKG checks whether the condition $\hat{e}\left(P,V\right)==\hat{e}\left(W_{1},W\right)\hat{e}\left(mpk,W_{2}\right)$ is fulfilled in order to accept the communication. The justification of that condition is explained below:
$\hat{e}\left(P,V\right)$
= $\hat{e}\left(P,T+D_{ID_{2}}\right)$
= $\hat{e}\left(P,\left(r+D_{ID_{1}}\right)\cdot W+D_{ID_{2}}\right)$
= $\hat{e}\left(P,\left(r+l+h\cdot msk\right)\cdot W+msk\cdot Q_{ID_{2}}\right)$
= $\hat{e}(P,\left(r+l\right)\cdot W+msk\cdot \left(h\cdot W+Q_{ID_{2}}\right)$
= $\hat{e}\left(P,\left(r+l\right)\cdot W\right)\cdot \hat{e}\left(P,msk\cdot \left(h\cdot W+Q_{ID_{2}}\right)\right)$
= $\hat{e}\left(P\cdot \left(r+l\right),W\right)\cdot \hat{e}\left(P\cdot msk,h\cdot W+Q_{ID_{2}}\right)$
= $\hat{e}\left(P\cdot r+P\cdot l,W\right)\cdot \hat{e}\left(mpk,h\cdot W+Q_{ID_{2}}\right)$
= $\hat{e}\left(U_{1}+Q_{ID_{1}},W\right)\cdot \hat{e}\left(mpk,W_{2}\right)$
= $\hat{e}\left(W_{1},W\right)\cdot \hat{e}\left(mpk,W_{2}\right).$
- Session Key Generation: When both server and client have been mutually authenticated, the session key (see Figure 7) is generated, at the same time, on the client side and on the server side. Afterwards, the exchanged messages are encrypted using the produced session key. In particular, the communication exchange between server and doctor is performed using the stream cipher SNOW 3G [42] using the obtained session key.

## 6. Security Analysis

This section includes a brief review on the protection provided by the system against different types of attacks.

A spoofing attack and/or cloning of the card would be hardly successful in the proposal, since these types of attacks involve the generation of the HMAC, but this generation requires the server master private key, the ID of a registered medical practitioner and the patient record number. Even if an external attacker obtains this information, the combination between the physical identifier of the NFC wristband and the patient record number is unique.

If the attacker corrupts the data of the wristband, the system can easily detect it because the server can know that the information used by the attacker does not coincide with the stored data. Thus, one of the strong points of the proposal is that the data saved on the NFC wristbands are not sensitive data.

If an attacker wants to emulate the wristband with an Android device, the attack is detected because the application is restricted only to read passive NFC tags.

Denial of Service attacks based on overloading the server with a large number of false requests are restricted because only those requests associated to the ID of a registered medical practitioner will take effect. Once the corresponding private key is assigned, additional requests from the same number will not be attended.

Regarding Man in the Middle attacks, they would be easily detectable because the number of members who are allowed to make requests to the server is limited to those who are working at the time of the request.

Regarding ESL attacks, the corresponding security level is based on the primitive of the ESL-secure ID-AKE protocol for mobile client-server environments included in the scheme proposed. In addition, there is a client-to-server authentication that prevents an adversary can impersonate a legitimate medical staff person to share information with the server through the use of the ECDH problem. In the case of the server-to-client authentication, the same reasoning is applicable. The protection is provided through a mutual authentication scheme and a SNOW 3G stream cipher with shared secret key obtained by an ECDH scheme. Apart from this, a key agreement procedure is defined to obtain the key required to encrypt all the communications between the server and the clients through the same stream cipher. This key agreement provides protection under known-session-key attacks. Finally, an implicit implicit key confirmation is used based on a random oracle model, in order to offer partial forward secrecy in this model.

The system design includes two servers, the intermediate server and the second server with the private key generator. The use of two different physical servers is proposed to have an additional security layer for key management. In this way, firewalls can be added independently to each server, and different secure rules can be applied in the communications between them. In other words, the communication of the private key generator with the Extranet might be denied, and just some interactions with the intermediate server might be allowed through, for example, an Intranet. Furthermore, if the intermediate server is corrupted by an attacker, the key generator will not be affected.

## 7. Performance Analysis

**Figure 8:**
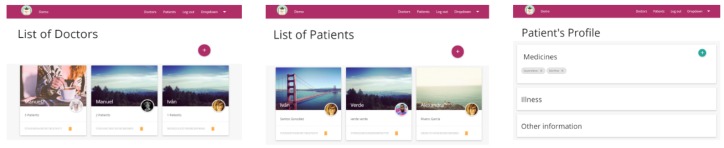
Web application.

Some basic prototypes of the proposal have been implemented on the client side, and the implementation is mainly formed by a web and a mobile application. On the server side, the prototype contains a data server and the private key generator. The web application is in charge of the management of patients’ and doctors’ information, and of the assignment of doctors to patients (see Figure 8).

**Figure 9:**
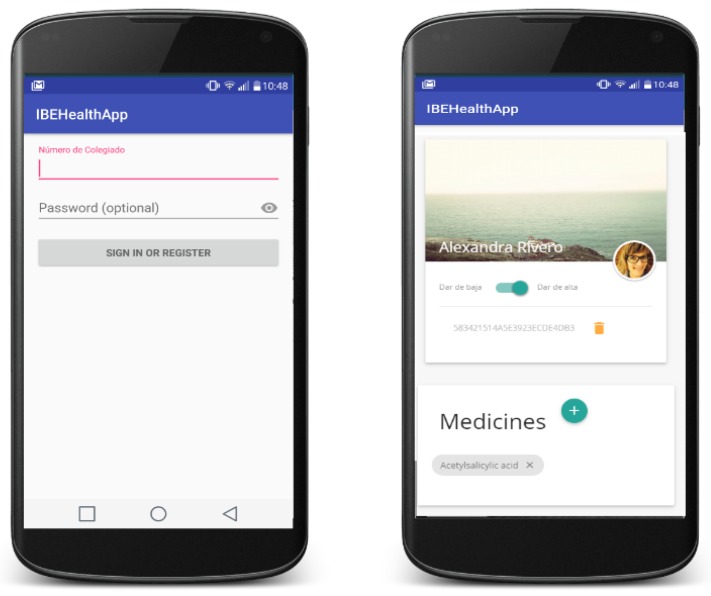
Mobile application. (**left**) View of registration; (**right**) View of patient data.

Thanks to the mobile application, doctors can easily identify and analyse the patient record. The integration of the application with the NFC sensor of the smartphones has been implemented for the Android platform in order to read and write NFC tags in a secure way (see Figure 9).

The data model for patient information is based on the clinic history containing is information related with affiliation data and health care data. Among the information related to the health, medical staff can analyse: reason for consultation (or hospitalization), personal history (allergies, habits, medical history, surgical history, family history, social history or current treatment), family background, current illness, anamnesis by organs, physical exploration results, differential diagnosis, supplementary tests, diagnostic trial and even the current therapeutic plan.

In the back-end side, the server was implemented based on a Model-View-Controller design pattern and with a non-relational database. On the one hand, the public access to the server was limited to restrict the access to the PKG, generated by a REpresentational State Transfer (REST) API. On the other hand, the internal communication between the server and the PKG is generated by a local REST API that has external restrictions, which means that a query can be generated only in the local site of the server.

The prototypes have undergone some tests. Since the HMAC generation depends on the server computer capabilities, different traces of the communication system were collected and evaluated. During these tests, the intermediate server was a computer with a quad core processor (Intel(R) Core(TM) i7-3537U CPU @ 2000 GHz) (Intel Corporation, Santa Clara, CA, USA), 4 GB of RAM memory, 1 TB of storage memory and the Windows 10 Pro version (×64 bits). This computer was used to access to the web application (see Figure 8) and patients’ data. The private key generator was a similar computer, with an Intel i7-4702MQ processor (Intel(R) Core(TM) CPU @ 2000 GHz) (Intel Corporation, Santa Clara, CA, USA), 8 GB of RAM memory, 1 TB of storage memory and Windows 10 operating system version (×64 bits).

As the client, a Samsung Galaxy S6 (Samsung Electronics, Suwon, Korea) with Exynos 7420 octa core processor (Samsung Electronics, Suwon, Korea) (4 × 2.1 GHz Cortex-A57 4 × 1.5 GHz Cortex-A53), 3 GB of RAM memory, 32 GB of storage memory and the 6.0 Android version was used. In these experiments, an amount of 100 data packets were collected to be analysed in order to obtain results related with the overall response time.

The network used to perform all the tests was made up of a Tp-link TL-WR841N Wi-Fi router that theoretically offers a maximum transfer rate of 300 Mbps and an Internet connection of 100 Mbps. All communications were tested in the laboratory with the prototypes, obtaining a transmission time of less than 1 s. Taking these results into account, it may be stated that less than a minute is necessary both to assign a NFC wristband to a patient and to read patients’ data.

Apart from implementing different parts of the proposal to show its feasibility, several simulations of its behaviour in a real environment have been done to measure some parameters that indicate improvements with respect to the current system.

One of the most important points of this proposal is to save time on the tasks of the medical staff. Currently, before assisting a patient, the doctor must review the patient record, which, in many cases, is printed and located in specific areas.

The mobility feature of the devices used in the proposed system allows for reducing the time spent by medical staff on roaming because they will not need to go to the documentation area and so they can attend patients without limitations.

**Figure 10:**
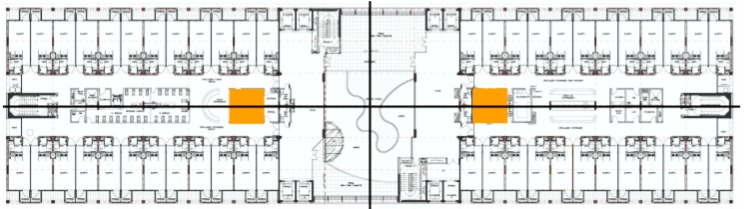
Hospital map.

Some simulations on the distribution of a floor in a real hospital (see Figure 10) have been performed. In this example, the orange zones are the areas where the hospital has the documentation areas for the doctors.

**Figure 11:**
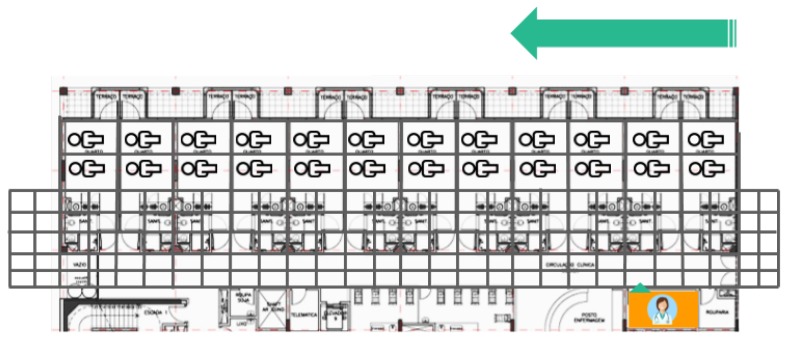
Grid map of the simulated area.

For the simulations, the map of the floor was divided into four parts so that each one was run in a quarter of the map. A grid map of each part was generated to evaluate the route that doctors follow to visit each patient (see Figure 11). The best route in the current system consists of visiting patients located in adjoining rooms. In the simulation, it is assumed that there are two patients in each room.

**Figure 12:**
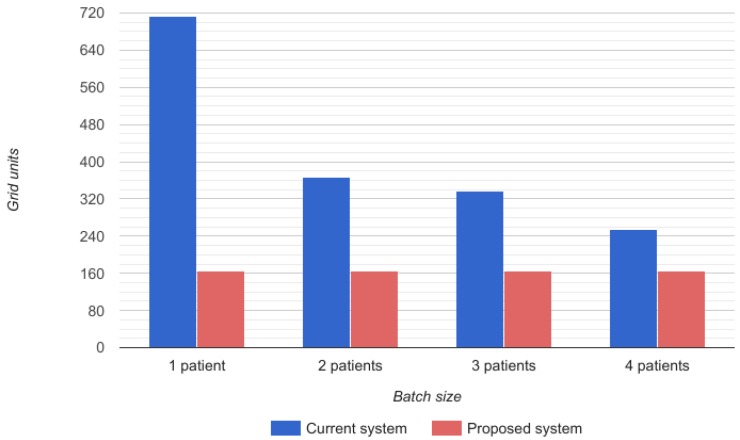
Time required for a doctor’s route.

The time required for the doctor’s route depends on the number of patients that a doctor can visit at once, that is to say, the size of the batches of patients that he/she has to visit before going back to the documentation area. In the proposed system, the time required for the doctor’s route is always constant because it is assumed that he/she does not have to go to the documentation area. In Figure 12, a representation of the grid units covered by the physician can be observed. Blue bars reflect the grids covered in the current system while red ones show those required by the proposed system.

The improvement observed in the time consumed by each doctor in the route to attend patients in batches is increased by the saving on the time that the identification of patients requires since, with the proposed system, this identification is automatic thanks to the mobile devices of doctors and NFC wristbands of patients.

## 8. Conclusions

The identification of patients in emergency and hospitalization services is a major problem in the healthcare sector. The secure and efficient management of patient records is another key point. These two issues are addressed in this work through the proposal of a distributed framework for the secure management of patients’ information.

The proposed system is based, on the one hand, on NFC wristbands assigned to patients and mobile devices assigned to medical staff, and, on the other hand, on two servers to manage patients’ data and to generate private keys separately.

A modification of an ID-based Authentication and Key Exchange Protocol resistant to Ephemeral-Secret-Leakage attacks for mobile devices is presented in this paper to provide mutual authentication between server and health staff. Specifically, the proposed protocols include client-to-server authentication, server-to-client authentication, key agreement, implicit key confirmation and secure channel to share keys.

This is part of a work in progress. All the curves used in the performed beta implementation were chosen according to the National Institute of Standards and Technology suggestions [40] over $F_{p}$, but in the next implementations, new curves over $F_{p}^{2}$, and in particular the new curve called FourQ, will be used to try to improve efficiency [43] in the implementation of the ECDH protocol on the most used processors in current smartphones. Furthermore, a future version of the system will include a robust and secure anonymity scheme based on pseudonyms. The issue of non-traceability of patients is also an open issue.

Finally, although some simulations were generated and the system was tested in the laboratory with some medical staff, in the immediate future, the system will be deployed in a real hospital to analyse the real improvements contributed by this proposal compared with the traditional method.
